# Efficacy and safety of lenalidomide in HIV-associated cryptococcal meningitis patients with persistent intracranial inflammation: an open-label, single-arm, prospective interventional study

**DOI:** 10.1186/s12974-023-02717-w

**Published:** 2023-02-15

**Authors:** Zhikai Wan, Ran Tao, Jiangjin Hui, Xiang Liu, Xiaorong Peng, Yongzheng Guo, Xueling Zhu, Ying Huang, Biao Zhu

**Affiliations:** The Department of Infectious Diseases, State Key Laboratory for Diagnosis and Treatment of Infectious Diseases, National Clinical Research Center for Infectious Diseases, Collaborative Innovation Center for Diagnosis and Treatment of Infectious Diseases, The First Affiliated Hospital, School of Medicine, Zhejiang University, Hangzhou, China

**Keywords:** HIV-associated cryptococcal meningitis, Lenalidomide, Immunomodulatory drug, Persistent intracranial inflammation

## Abstract

**Background:**

Patients with human immunodeficiency virus-associated cryptococcal meningitis (HIV-CM) have persistent intracranial inflammation despite negative cerebrospinal fluid (CSF) fungal cultures after optimal treatment for CM, which could be devastating for the central nervous system. However, a definitive treatment strategy for persistent intracranial inflammation despite optimal antifungal therapies is undefined.

**Methods:**

We identified 14 HIV-CM patients with persistent intracranial inflammation and conducted a 24-week, prospective, interventional study. All participants received lenalidomide (25 mg, p.o.) on days 1 to 21 of a 28-day cycle. Follow-up lasted for 24 weeks with visits at baseline and weeks 4, 8, 12, and 24. The primary endpoint was the change in clinical manifestations, routine CSF parameters, and MRI findings after lenalidomide treatment. An exploratory analysis was made on changes in cytokine levels in CSF. Safety and efficacy analyses were undertaken in patients who received at least one dose of lenalidomide.

**Results:**

Of 14 participants, 11 patients completed the 24 weeks of follow-up. Rapid clinical remission following lenalidomide therapy was observed. Clinical manifestations (fever, headache, altered mentation) were reversed fully by week-4 and remained stable during follow-up. A significant reduction in white blood cell (WBC) count in CSF was noted occurred at week-4 (*P* = 0.009). The median protein concentration in CSF decreased from 1.4 (0.7–3.2) g/L at baseline to 0.9 (0.6–1.4) at week-4 (*P* = 0.004). The median albumin concentration in CSF decreased from 79.2 (48.4–149.8) mg/L at baseline to 55.3 (38.3–89.0) mg/L at week-4 (*P* = 0.011). The WBC count, protein level, and albumin level in CSF remained stable and approached a normal range through week-24. There was no significant change in immunoglobulin-G, intracranial pressure (ICP), or chloride-ion concentration at each visit. Brain MRI demonstrated multiple lesions to be absorbed post-therapy. Levels of tumor necrosis factor-α granulocyte colony stimulating factor, interleukin (IL)-6, and IL-17A decreased significantly during 24-week follow-up. Two (14.3%) patients had mild skin rash, which resolved spontaneously. Lenalidomide-related serious adverse events were not observed.

**Conclusion:**

Lenalidomide could improve persistent intracranial inflammation in HIV-CM patients significantly and was well tolerated without serious adverse events observed. And the additional randomized controlled study is required to further validate the finding.

**Supplementary Information:**

The online version contains supplementary material available at 10.1186/s12974-023-02717-w.

## Introduction

Cryptococcal meningitis (CM) is a type of invasive infectious disease caused by the fungus *Cryptococcus neoformans*. CM occurs primarily in immunocompromised hosts, such as people living with the human immunodeficiency virus (PLHIV) [1]. CM is a subacute meningoencephalitis, but its pathogenicity increases in PLHIV, eventually becoming life-threatening [2]. It has been reported that CM is a major cause of death in people with advanced human immunodeficiency virus (HIV) infection, especially those living in Africa and southeast Asia [3]. Fatalities from HIV-associated cryptococcal meningitis (HIV-CM) number ~ 180,000 deaths/year worldwide, accounting for 15% of HIV-related deaths [4].

Recently, the mortality of patients with HIV-CM has decreased significantly thanks to active antifungal therapy, aggressive management of increased intracranial pressure (ICP), and timely antiretroviral therapy (ART) initiation [5]. After long-term efficacious antifungal treatment (e.g., amphotericin B, flucytosine, fluconazole) and ART, the cryptococcus culture in cerebrospinal fluid (CSF) becomes negative and the HIV-RNA in plasma becomes undetectable in CM patients. However, some of these patients continue to have abnormal CSF parameters and clinical symptoms persist or even worsen [6]. Their clinical features are similar to those caused by encephalitis syndrome, and the clinical manifestations are mainly fever, headache, seizures, and even cognitive dysfunction. Simultaneously, imaging shows aberrant leptomeningeal enhancement, new parenchymal brain lesions, and white matter lesions [7, 8].

Studies have shown that the phenomena described above occur because of brain injury mediated by persistent intracranial inflammation [9]. Despite ART, residual low levels of viral replication from latent reservoirs of HIV molecules and the subsequent antiretroviral immune response may be the cause of sustained immune activation in the central nervous system (CNS) [10]. Subsequently, the positive feedback loop of immune-mediated tissue damage and repair processes can lead to persistent, chronic inflammation in the CNS [9]. On the one hand, chronic CNS inflammation can induce neuronal insults, leading to cognitive dysfunction [11]. On the other hand, persistent neuroinflammation leads to mis-migration of immune cells and increased permeability of blood–brain barrier (BBB), resulting in additional damage to surrounding tissues which in turn causes continual inflammation. Vicious cycle of continual inflammation, blood–brain barrier permeability and tissue damage all contributing to an increase in intracranial pressure (ICP), thereby increasing the risk of seizures and brain herniation [12]. In previous study, the chemokine and cytokine cascade caused by skewing of the Th1–Th2 balance and reduced CD4 + T-lymphocytes count were found to be important contributors to high intracranial pressure (HICP) [13]. Persistent ICP elevation is the most accurate predictor of poor prognosis in patients with HIV-CM [14]. Lumbar puncture and ventriculoperitoneal shunts (VPS) are important management strategies for high ICP patients. However, the use of VPS in HIV-infected patients is debatable given the risk of shunt infection [15]. Therefore, safe and efficacious interventions are needed urgently for these patients to manage persistent intracranial inflammation to improve clinical outcomes.

Mechanistic studies have suggested that the persistent intracranial inflammation in CM is characterized by high levels of T helper 1 (Th1) cell-associated proinflammatory cytokines such as interferon (IFN)-γ, tumor necrosis factor (TNF)-α, granulocyte colony-stimulating factor (G-CSF), and interleukin (IL)-6 in CSF [16, 17]. Thus, anti-inflammatory or immunomodulatory methods have become the primary therapeutic options for persistent intracranial inflammation. Several case reports have documented neurological improvement after use of corticosteroids, hydroxychloroquine, thalidomide, and adalimumab [18]. However, due to a lack of data from prospective clinical trials and the small number of patients treated, judging the efficacy and safety of the immune-system modulators stated above is not possible [19].

The TNF-α antagonist lenalidomide has been shown to exert immunomodulatory and anti-inflammatory effects, and is a widely used and efficacious anti-tumor agent [20, 21]. As an analog of thalidomide, lenalidomide is a potent immune-system modulator with few side-effects [22]. In animal models, lenalidomide has been shown to cross the BBB, and use as an adjuvant treatment for tuberculous meningitis has been attempted [23]. Due to its low neurotoxicity, scholars have tried to use lenalidomide against meningeal myelomas and CNS lymphomas [24, 25].

We conducted a prospective interventional clinical trial to determine if lenalidomide improved the clinical outcomes of HIV-CM patients suffering from persistent intracranial inflammation. We assessed the clinical features of enrolled individuals in terms of clinical presentations, laboratory features, and neuroimaging characteristics, and we evaluated clinical outcomes after 6 months of follow-up. In addition, the cytokine concentrations in the CSF of participants were measured and compared before and after lenalidomide therapy.

## Methods

### Ethical approval of the study protocol

The trial protocol (and other documents) was approved by the Ethics Committee of the First Affiliated Hospital of Zhejiang University (Zhejiang, China) in accordance with the Helsinki Declaration 1964 and its later amendments. All patients received detailed information about the proposed treatment option. Written informed consent was obtained from all patients. This study is registered with the China Clinical Trial Registry (www.chictr.org/cn/; ChiCTR1900023184).

### Study design and participants

This was an open-label, single-arm, clinical trial conducted in the National Clinical Research Center for Infectious Diseases (Zhejiang, China). The primary inclusion criterion was HIV-CM patients with a positive diagnosis of CNS inflammation despite accepting ART and successful induction therapy for CM (Additional file 1 reveals the full inclusion and exclusion criteria). “Successful induction therapy” was defined as a substantial improvement in clinical manifestations and cryptococcal cultures in CSF were negative on two or more occasions after standard induction treatment [26]. Patients with severe myelosuppression, severe cardiovascular/cerebrovascular diseases, or liver/kidney dysfunctions were excluded.

### Procedures

**Fig. 1:**
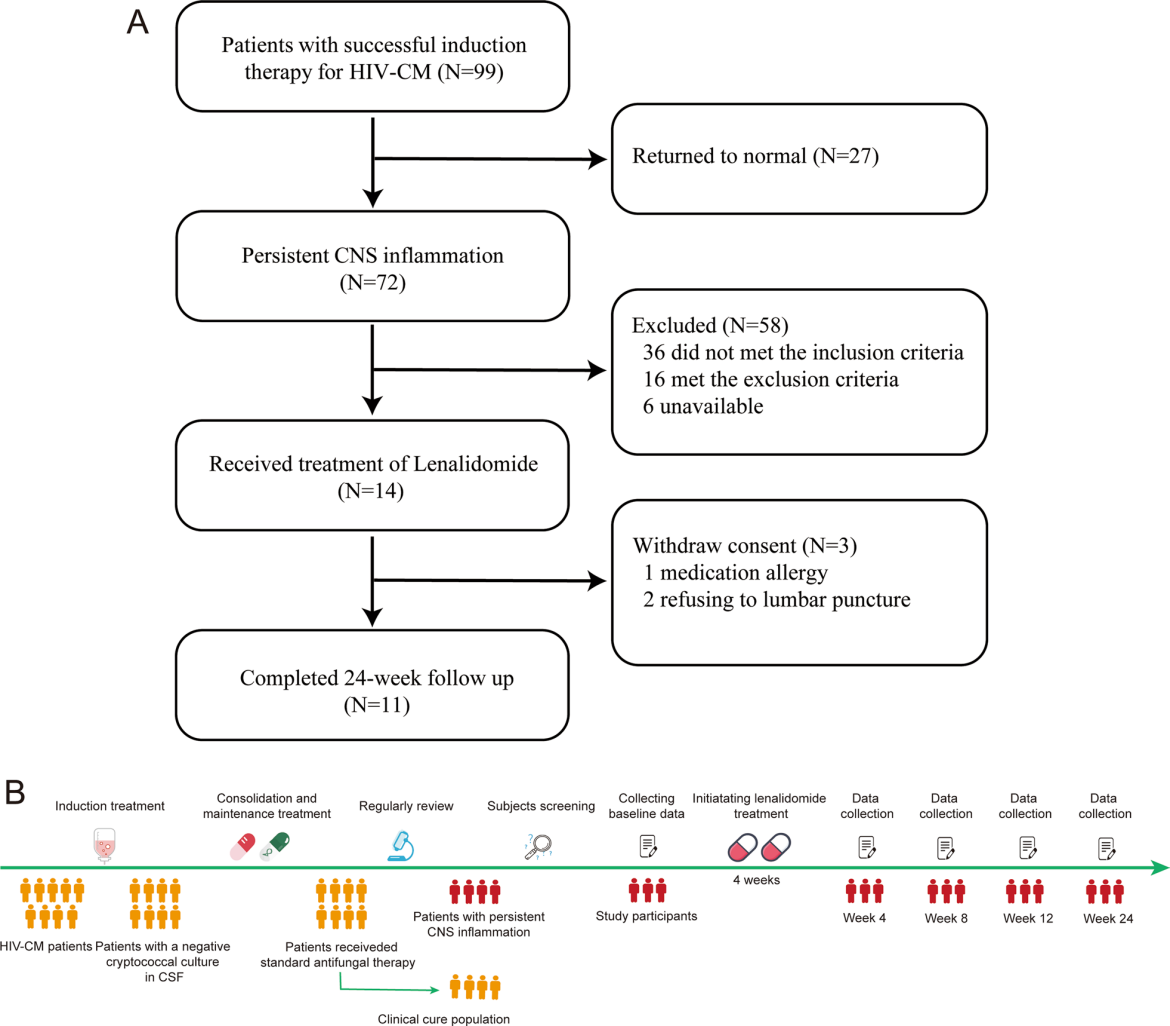
Profile of the clinical trial. **A** The screening process of the study population; **B** study screening time point, procedures, enrollment and follow-up. Patients enrolled in this study were HIV-1-infected individuals with CM. All patients had already completed initial therapy of CM with amphotericin B (AmB) and 5-flucytosine (5-FC) and had negative CSF fungal cultures; they continued consolidation treatment and maintenance treatment with fluconazole (FLU). Received standard antifungal and antiretroviral therapy, these patients had a negative cryptococcal culture in CSF. However, CSF indices and neuroimaging characteristics remained abnormal, and clinical manifestations did not improve significantly (or even worsened), which was thought to be attributed to persistent intracranial inflammation. Based on the inclusion and exclusion criteria, we screened the patients and finally tried to treat them with lenalidomide. Final participants received five visits over 24 weeks (baseline as well as at weeks 4, 8, 12, and 24). At these visits, we assessed clinical symptoms and adverse reactions, and undertook blood tests, clinical data and sample collection. CSF: cerebrospinal fluid; CNS: central nervous system; HIV-CM: HIV-associated cryptococcal meningitis

This was a single-arm prospective study with all data were collected prospectively. All participants received lenalidomide (25 mg, p.o.) on days 1 to 21 of a 28-day cycle to avoid severe myelotoxicity. Treatment and observation lasted 6 months. Before enrollment, eligible individuals were screened in addition to undergoing laboratory tests, and a comprehensive medical history was taken. Subjects who met the inclusion criteria but not the exclusion criteria were enrolled in the study. The time point at which lenalidomide treatment was planned to be initiated was considered to be the baseline time (Fig. 1B). We scheduled all participants to receive five visits over 24 weeks (baseline as well as at weeks 4, 8, 12, and 24). At these visits, we assessed clinical symptoms and adverse reactions and undertook blood tests (full blood count (FBC), liver/kidney function tests, coagulation function, C-reactive protein, cluster of differentiation (CD)4^+^ T-lymphocyte counts, HIV load). HIV-RNA was measured by TaqMan 2.0 (Roche, Basel, Switzerland), which had a lower limit of quantification of 20 copies/mL. Furthermore, at each visit, CSF samples were collected by lumbar puncture to measure CSF parameters and immunological indices. Information regarding neuroimaging characteristics was collected at screening as well as at weeks 8 and 24 (Additional file 1: Table S1).

### Evaluation of clinical efficacy and safety

The primary endpoint of this pilot study was the change in clinical manifestations of participants, routine CSF parameters, and imaging findings after lenalidomide treatment. Routine CSF parameters were cell counts as well as levels of protein and glucose. The response for lenalidomide therapy based on imaging was assessed by magnetic resonance imaging (MRI) of the brain. Clinical data, routine CSF parameters, and neuroimaging information were compared between values at baseline and 4, 8, 12, and 24 weeks post-treatment. Drug safety was evaluated mainly through adverse events, vital signs, and laboratory parameters. At each visit, vital signs were assessed and height and weight measured; laboratory tests for FBC, biochemistry, coagulation function, and CD4^+^ T-lymphocytes as well as counts for other immune-cell subsets were taken. Demographic data (age, sex), medical history, laboratory parameters, and MRI findings were obtained from medical records or hospital databases.

### Detection of proinflammatory cytokines in CSF

CSF samples were collected from participants. They were centrifuged immediately and the supernatant was stored at − 80 °C until testing. The concentration of cytokines in blood was determined by a magnetic bead-based multiplex panel (Luminex™; Bio-Rad Laboratories, Hercules, CA, USA). In 14 patients, levels of the following cytokines in CSF were evaluated: TNF-α, IFN-γ, IL-1α, IL-1β, IL-2, IL-3, IL-4, IL-6, IL-8, IL-10, IL-12p40, IL-12p70, IL-13, IL-17A, interferon-gamma induced protein 10, monocyte chemotactic protein-1, macrophage inflammatory protein (MIP)-1α, MIP-1β, G-CSF, granulocyte–macrophage colony-stimulating factor, soluble CD40 ligand, complement C3, complement factor H (CFH), fibroblast growth factor-2, platelet-derived growth factor-AA, growth-related oncogene, alpha 2-macroglobulin (α2M), apolipoprotein AI (Apo AI), apolipoprotein E (Apo E), amyloid beta 1–42, total tau, and phosphorylated tau. Cytokine concentrations were expressed in pg/mL and determined before and after lenalidomide therapy (refer to Additional file 1: Table S2 for explanation of abbreviations).

### Statistical analyses

Demographic and laboratory data are expressed as median (interquartile range (IQR)) for continuous variables. Categorical variables are described as numbers and percentages. Efficacy and safety endpoints are reported based on the full-analysis set, which consisted of the data of all patients who received at least one dose of lenalidomide. Clinical and imaging data were recorded in Excel™ 2019 (Microsoft, Redmond, WA, USA). The Wilcoxon matched-pair signed-rank test and McNemar test were applied to compare baseline and post-treatment values. *P* < 0.05 (two-sided) was considered significant, and P-values were adjusted for multiple comparisons via a segmentation method. Statistical analyses were undertaken with SPSS 23 (IBM, Armonk, NY, USA). Each parameter was tested for a normal distribution with SPSS 23. Analyses of enrichment of genes of cytokines were undertaken using the Gene Ontology (GO) database (www.geneontology.org/). Charts were generated using Prism 8 (GraphPad, San Diego, CA, USA).

## Results

### Characteristics of participants

Between January 2017 and October 2020, we screened 99 patients who had successful induction therapy for HIV-CM, 72 of whom had developed CNS inflammation post-ART. Finally, 14 patients were found to be eligible for our study on the basis of our inclusion and exclusion criteria (Fig. 1). One individual withdrew his consent to participate due to allergy to lenalidomide. Of the 13 participants who completed six cycles of therapy, two participants declined to undergo lumbar puncture during follow-up due to personal reasons, and so were excluded from analyses of CSF biomarkers.

All participants were Asian men of median age 32.50 (IQR, 30.5–39.3) years. At baseline, the median time from the CM diagnosis to ART initiation was 25.5 (18.5–36.5) days. The median time from ART initiation to lenalidomide treatment was 558 (314.0–882.3) days. The median CD4^+^ T-lymphocyte count was 151.5 (117.0–342.3)/µL. For 11 participants, the HIV-RNA load in plasma was below the limit of detection of the assay. The predominant clinical manifestations were fever (four cases, 28.6%), headache, (seven, 50%) altered mentation (four, 28.6%), and seizures (two, 14.3%) (Table 1, Additional file 1: Table S3).

**Table 1:** Characteristics of participants

Characteristic	
Age (year)	32.5 (30.5–39.3)
Sex, number (%)	
Male	14(100)
Female	0 (0)
Ethnic group, number (%)	
Asian	14 (100)
Other	0 (0)
Body mass index (kg/m^2^)	23.1 (20.2–26.4)
Time from CM to ART initiation, days	25.5 (18.5–36.5)
Time from ART initiation to LEN, days	558.0 (314.0–882.3)
Time from anti antifungal to LEN, days	581.0 (340.5–906.5)
ART regimen, number (%)	
2NRTI + 1NNRTI	2 (14.3)
2NRTI + 1PI	1 (7.1)
2NRTI + 1 INSTI	11 (78.6)
CD4 + T-lymphocytes count/µL	151.5 (117.0–342.3)
HIV-RNA, number (%)	
< 50 copies/ml	11 (78.6)
50–200 copies/ml	0
> 200 copies/ml	3 (22.4)
CD4^+^ T-lymphocyte count (screen), number (%)	
< 200 cells/ul	9 (64.3)
200–500 cells/ul	5 (35.7)
> 500 cells/ul	0 (0)
Clinical presentation, number (%)	
Fever	4 (28.6)
Headache	7 (50)
Altered mentation	4 (28.6)
Seizures	2 (14.3)

Data are median (IQR) or *n* (%)

ART: antiretroviral therapy; CM: cryptococcal meningitis; LEN: lenalidomide; NRTI: nucleoside reverse transcriptase inhibitor; NNRTI: non-nucleoside reverse transcriptase inhibitor; INSTI: integrase strand transfer inhibitor; PI: protease inhibitor

### Rapid clinical remission following lenalidomide therapy

All participants received at least one cycle of lenalidomide. When all participants had completed the first cycle of lenalidomide therapy (week-4), clinical presentations such as fever, headache, and convulsions had reversed fully. Only one (7.1%) patient continued to show altered mentation but had recovered by week-12 (Table 2).

**Table 2:** Rapid clinical remission of participants following lenalidomide therapy

Week	0	4	8	12	24
Fever, number (%)	4 (28.6)	0 (0.0)	0 (0.0)	0 (0.0)	0 (0.0)
Headache, number (%)	7 (50.0)	0 (0.0)	0 (0.0)	0 (0.0)	0 (0.0)
Altered mentation number (%)	4 (28.6)	1 (7.1)	1 (7.1)	0 (0.0)	0 (0.0)
Seizures, number (%)	2 (14.3)	0 (0.0)	0 (0.0)	0 (0.0)	0 (0.0)

### Rapid reduction in routine CSF parameters following lenalidomide therapy

**Fig. 2:**
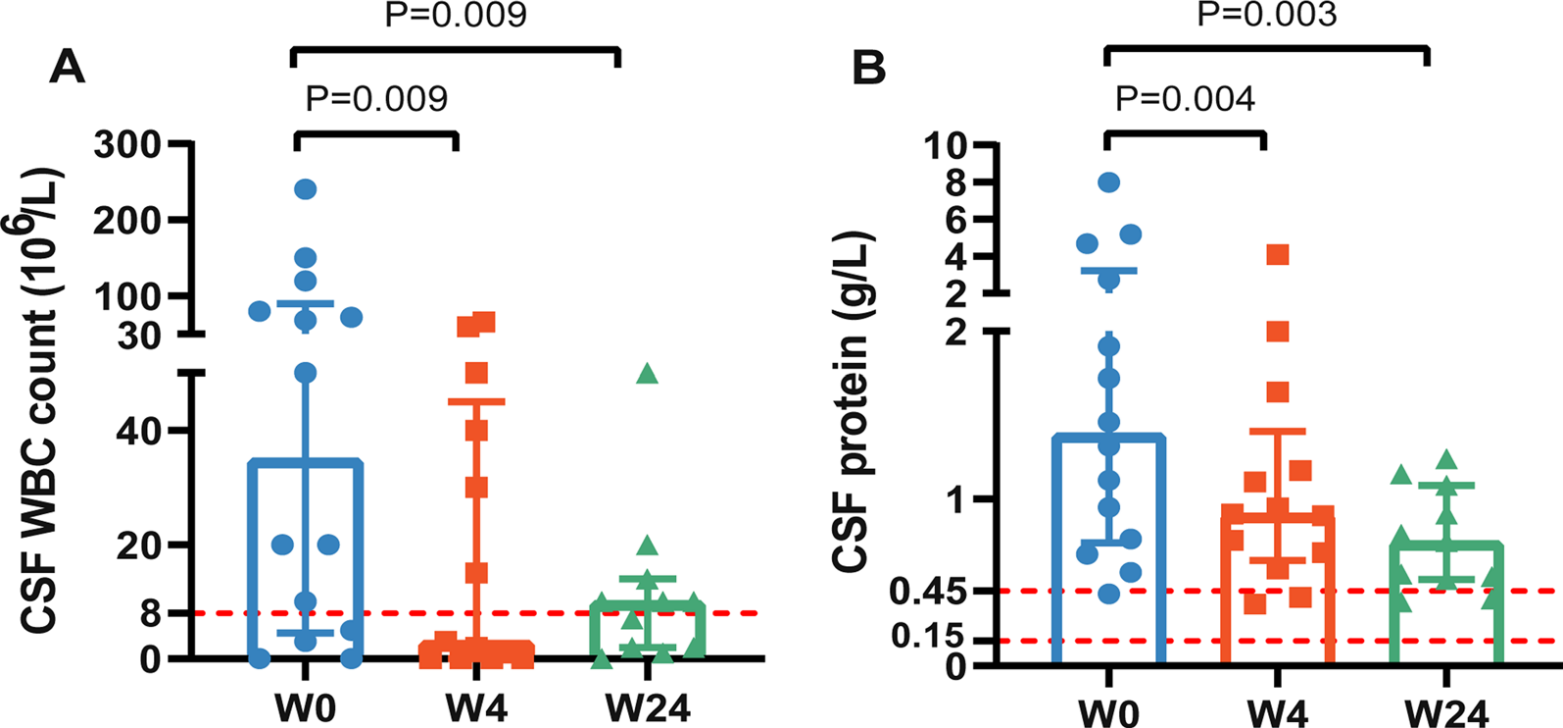
Change in CSF parameters at 24-week follow-up. A: Change in CSF WBC at 24-week follow-up. B: Change in CSF protein at 24-week follow-up. Rapid reduction in the WBC and protein level in CSF was observed following lenalidomide therapy. Red dotted lines represent the range of normal reference values for the WBC count and protein level in CSF. W: week. WBC: white blood cell; CSF: cerebrospinal fluid

A significant reduction in routine CSF parameters from those at baseline was observed (Fig. 2). The median WBC count in CSF at baseline was 35 × 10^6^/L (IQR, 4.5–90.0 × 10^6^/L). A significant reduction in the WBC count (× 10^6^/L) occurred at week-4 from baseline (week-4: 3.00 (IQR, 0–45.0) vs. week-0: 35 (IQR, 4.5–90.0), *P* = 0.009). Simultaneously, levels of protein and albumin in CSF decreased significantly in a short time. The median protein concentration in CSF decreased from 1.4 (IQR, 0.7–3.2) g/L at baseline to 0.9 (IQR, 0.6–1.4) g/L at week-4 (*P* = 0.004). The median albumin concentration in CSF decreased from 79.2 (48.4–149.8) mg/L at baseline to 55.3 (IQR, 38.3–89.0) mg/L at week-4 (*P* = 0.011). In CSF, the WBC count as well as levels of protein and albumin remained stable and approached the normal range through week-24 (WBC count = 10.0 (IQR, 2.0–14.0), *P* = 0.009; protein = 0.7 (IQR, 0.5–1.1) g/L, *P* = 0.003; albumin = 42.4 (IQR, 32.7–54.4) mg/L). After lenalidomide therapy for 24 weeks, the immunoglobulin-G level (mg/dL) in CSF was also decreased, though not significantly so (week-24: 11.2 (6.4–36.0) *vs*. week-0: 13.1 (IQR, 7.3–160.5), *P* = 0.263). However, there was a slight increase in ICP after 4 weeks of lenalidomide treatment, though the difference was not significant (160.0 (IQR, 144.0–220.0) *vs*. 150.0 (135.0–210.0), *P* = 0.248). There was no significant difference in ICP between week-24 and baseline. There was no significant change in CSF chloride-ion concentration at each visit, but the glucose level in CSF increased slightly (HR, 0.1; 95%CI 0–0.75, *P* = 0.044) at week-24 from that at baseline (Additional file 1: Table S4).

### Improvement in neuroimaging characteristics following lenalidomide therapy

The neuroimaging characteristics of all participants at baseline are summarized in Table 3. Lesions were located mainly in the meninges (10 cases, 71.4%), cerebral white matter (five, 35.7%), basal ganglia (four, 28.6%), and brain parenchyma (three, 21.4%). Ten (71.4%) patients showed inflammatory lesions, and other disorders included ischemia, encephalomalacia, and hemorrhage. Three (28.6%) patients had an unidentified lesion. An obvious remission in the inflammatory lesion was observed in eight (8/10, 80%) of patients after lenalidomide therapy (*P* = 0.008). Three (3/4, 75%) participants had an ischemic injury that improved and one (1/1 100%) patient had a hemorrhagic lesion that was absorbed.

**Table 3:** Neuroimaging characteristics of participants

Cranial MRI	Baseline	Week 24	P value*
Lesion location			
Meninges, number (%)	10 (71.4)	2 (14.3)	**0.008**
Brain parenchyma, number (%)	3 (21.4)	1 (7.1)	0.500
Cerebral white matter, number (%)	5 (35.7)	3 (21.4)	0.500
Basal ganglia, number (%)	4 (28.6)	3 (21.4)	> 0.999
Lesion nature			
Inflammation, number (%)	10 (71.4)	2 (14.3)	**0.008**
Encephalomalacia, number (%)	2 (14.3)	2 (14.3)	> 0.999
Hemorrhagic lesion, number (%)	1 (7.1)	0 (0)	> 0.999
Cerebral ischemia, number (%)	4 (28.6)	1 (7.1)	0.250
Unidentified lesion, number (%)	3 (21.4)	3 (21.4)	> 0.999

Data show that the number of lesions was not decreasing after 24 weeks of treatment with lenalidomide. *P value was determined by the McNemar test

### Significant decrease in levels of Th1 cell-associated proinflammatory cytokines

**Fig. 3:**
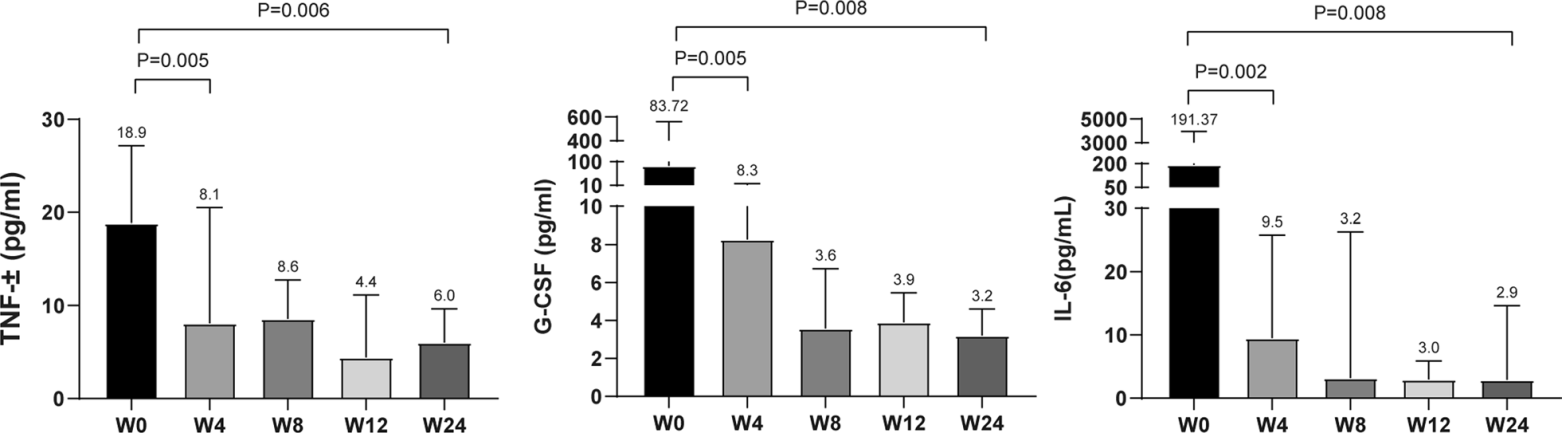
Th1 cell-associated proinflammatory cytokines. Concentrations of TNF-α, G-CSF, and IL-6 in the CSF from patients treated with lenalidomide decreased significantly during follow-up. W: week; TNF-α: tumor necrosis factor-α; G-CSF: granulocyte colony-stimulating factor; IL-6: interleukin-6

In an exploratory analysis, we looked for the most profound changes in cytokine concentrations in CSF. Patients had high levels of Th1 cell-associated proinflammatory cytokines, such as TNF-α, G-CSF, and IL-6, upon study enrollment. The median TNF-α level (pg/mL) decreased significantly from baseline to week-4 (18.9 (IQR, 4.5–27.2) to 8.1 (IQR, 4.1–20.5), *P* = 0.005), and its concentration tended to decline within 24 weeks. Levels of G-CSF and IL-6 in CSF were high. At baseline, the median level (pg/mL) of G-CSF and IL-6 was 83.7 (IQR, 3.9–562.2) and 191.4 (IQR, 3.9–3958.0), respectively. Compared with that at baseline, the median G-CSF level decreased by 90.1% at week-4 (week-4: 8.3 (IQR, 3.7–16.3) vs. week-0: 83.7 (IQR 3.9–562.2), *P* = 0.005) and decreased by 96.5% at week-24 (3.2 (IQR, 2.8–4.6), *P* = 0.008). The IL-6 level (pg/mL) decreased at week-4 from that at baseline, and remained diminished at week-24 (week-24: 2.9 (IQR, 2.8–14.7) *vs*. week-0: 191.4 (IQR, 3.9–3958.0), *P* = 0.008)) (Fig. 3). Levels of IL-17A, IL-10, α2M, Apo AI, CFH, and complement C3 decreased significantly during 24-week follow-up (Additional file 1: Figure S2). Analysis of enrichment of signaling pathways revealed that these cytokines were involved in “regulation of inflammatory response” and “regulation of the immune effector process” (Additional file 1: Figure S3).

### Safety analyses of lenalidomide

Safety analyses were undertaken in 14 patients who received at least one dose of lenalidomide. 1 patient withdraw from the study because of a grade II acute allergic reaction and 2 participants experienced grade I rash which resolved spontaneously. No Grade 3 or higher adverse events were observed during the 24-week follow-up period. Counts for WBCs, neutrophils, and platelets were reduced slightly after lenalidomide treatment. 2 WBC decreased (1 grade I and 1 grade II) and 4 low absolute neutrophil count (1 grade I and 3 grade II) was reported. The median WBC count (10^9^/L) dropped to its lowest value at week-12 (week-12: 4.1 (IQR, 3.0–4.9) *vs*. week-0: 6.8 (IQR, 5.7–8.1), *P* = 0.002) and the platelet count (10^9^/L) at week-8 (week-8: 173.0 (IQR, 130.0–189.5) *vs*. week-0: 260.0 (IQR, 209.0–299.0), *P* = 0.001), both of which remained within the normal IQR. Furthermore, a significant decrease in the neutrophil count (10^9^/L) was noted, which reached a minimum at week-12 (week-12: 1.2 (IQR, 0.9–1.6) *vs*. week-0 3.8 (IQR, 2.9–4.8), *P* = 0.002) (Additional file 1: Figure S4). However, the levels of the aforementioned indices at week-24 were increased to a certain extent and close to normal levels (Additional file 1: Table S5). Except for levels of blood urea nitrogen (BUN) and D-dimer, there was no obvious change in liver and kidney functions, blood lipid level, or coagulation function during follow-up. A slight increase in levels of BUN and total bilirubin at week-24 was noted, but they were within the normal range. A significantly lower level of D-dimer was noted at week-24 compared with that at baseline. Significant differences in the numbers of CD4^+^ T-lymphocytes and CD8^+^ T-lymphocytes were not observed after lenalidomide treatment.

## Discussion

We hypothesized that the immunomodulator lenalidomide could be considered as an alternative therapy for persistent intracranial inflammation in HIV-CM patients. As a novel immunomodulatory agent, lenalidomide was approved for the treatment of multiple myeloma and show anti-tumor activity in other hematological malignancies. In multiple clinical trials [27–29], lenalidomide was administered orally at a dose of 25 mg/day on days 1 to 21 of each 28-day cycle, which is recommended dosing regimen in the package insert. Considering that lenalidomide has never been applied in HIV-CM populations, in this study, patients received lenalidomide (25 mg orally, once per day) on days 1–21 every 28 days to avoid severe myelotoxicity. Within 3 weeks after lenalidomide treatment, symptoms of meningitis syndrome disappeared in most patients. The inflammation indices of CSF (WBC count, protein level, albumin level) also fell significantly and approached the normal range gradually. Changes in neuroimaging were observed among these patients, two of whom had obvious remission in the inflammatory lesion post-lenalidomide therapy, and other patients showed different degrees of neuroimaging improvement. In addition to observing rapid clinical remission following lenalidomide therapy, we found significant reductions in levels of Th1-cell associated proinflammatory cytokines such as TNF-α, IL-6, and G-CSF in CSF. Also, analysis of pathway enrichment using the GO database indicated that these cytokines were involved in “regulation of inflammatory response” and “regulation of immune effector process”.

Enrolled patients had CM and were infected with HIV-1. They all met the diagnostic criteria for HIV-CM and had received standard antifungal and timely antiretroviral therapy (two participants had started ART before the CM diagnosis) before enrollment. Among these patients (who had achieved successful induction therapy for CM), cryptococcal culture of CSF became negative and viral loads in plasma tended to be stable. The median interval between starting antifungal treatment and lenalidomide treatment was 581.0 (IQR 340.5–906.5) days. Long-term antifungal therapy did not improve persistent intracranial inflammation. Levels of CSF indicators continued to be abnormal, and clinical manifestations did not improve significantly (or even worsened). These phenomena were likely because of paradoxical immune reconstitution inflammatory syndrome (IRIS) [9, 30]. Participants had a high level of a Th1 cell-associated cytokine (TNF-α) and proinflammatory cytokines (G-CSF, IL-6, IL-17A) at baseline, data which are consistent with the literature [16, 31]. The consequences of an exaggerated Th1 cell-biased response are persistent malignant inflammation, BBB permeabilization, tissue damage, and increased ICP which, eventually, lead to cognitive dysfunction and even death [32].

Recently, it has been postulated that anti-inflammatory actions and protection by neurons are important against chronic CNS inflammation. In the past, corticosteroids have been used to treat persistent intracranial inflammation [33]. Guo and colleagues reported that in patients suffering from CM-IRIS without HIV infection, after using corticosteroids, headache was relieved immediately and the pressure elicited by CSF decreased within a short time [34]. Corticosteroids have been shown to be efficacious in HIV-infected individuals with CM-IRIS in some studies [35, 36]. However, cases of corticosteroid dependence have been reported, whereby inflammation relapses usually occurred if the corticosteroid dose was tapered or interrupted [37]. In addition, corticosteroid use in immunocompromised patients can increase the risk of infection and death [38]. Furthermore, other immunomodulators have been tested in severe cases of chronic inflammation that mediates brain injury. In HIV-infected patients with corticosteroid-dependent and life-threatening CM-IRIS, thalidomide administration was shown to induce rapid clinical remission [37]. Anti-inflammatory strategies using minocycline and selegiline have been tested in several clinical trials, but none exerted a significant clinically beneficial effect [39]. A recent study, however, demonstrated that intranasal-delivered insulin could reverse neuronal injury. This phenomenon may be related to a reduction in microglia activation and suppression of chronic inflammation, and is now being tested in PLHIV [40, 41].

Lenalidomide is thought to have more powerful immunomodulatory functions, but fewer side-effects, than thalidomide [42]. Different from previous reports using thalidomide [37], the treatment cycle of lenalidomide was 6 months and was intermittent in the present study. In addition to observing rapid clinical remission following lenalidomide therapy, we found significant reductions in levels of proinflammatory cytokines such as TNF-α, IL-6, and G-CSF in CSF. Thus, lenalidomide may inhibit the intense Th1 cell-based proinflammatory response in the brain, thereby improving clinical symptoms. Furthermore, compared with corticosteroids and thalidomide, lenalidomide seems to be safe and well-tolerated [12]. In our study, severe adverse events were not observed during follow-up, and only two patients had mild skin rash. Compared with values at baseline, major changes in routine blood parameters or indicators of liver/kidney function were not observed during follow-up.

There are several limitations to our study. The principal limitation is the small sample single-arm design, it is possible confounding of time may have occurred, which prevents us from drawing definitive conclusions. We did this because identifying a suitable control for a randomized study is challenging with no other approved treatment options for this patient population in China in 2017. It’s difficult to ethically randomize them to a control group. In the past, corticosteroids have been tried to treat these patients, but serious adverse effects including severe infection, osteoporosis, and obesity have given us pause [12]. Patients in this study were chosen for lenalidomide therapy based on disease progression after exhaustion of all other treatment options, and we found the clinical symptoms, examination indexes and head MRI of the patients were remarkably improved after treatment. Nevertheless, the study population was mainly middle-aged Chinese men with HIV-CM, which prevented extrapolation of our findings to other populations. Third, due to a lack of effective quantitative tools, MRI information was not displayed fully (though neuroimaging findings of most patients during follow-up were accessible). With regard to these, a reasonable, scientific, prospective, multicenter, randomized controlled study is required to further determine the anti-inflammatory effects of lenalidomide.

## Conclusions

The findings of this study demonstrated that lenalidomide was safe to use, well-tolerated, and there may be clinical benefit of lenalidomide in HIV-CM patients who develop persistent intracranial inflammation. Furthermore, lenalidomide exerts an immunomodulatory effect upon proinflammatory cytokines in CSF. Given the close association between cytokines and neuroinflammation [12], lenalidomide could be an alternative therapeutic strategy in HIV-CM patients with persistent intracranial inflammation. And a further prospective, randomized controlled study is needed to elucidate this issue.

## Supplementary Information

**Additional file 1. **Definition of primary HIV infection. Diagnostic criteria for HIV-CM. Inclusion criteria. Exclusion criteria. **Table S1.** Follow-up procedures and items for the clinical trial. **Table S2.** Abbreviations for cytokines in CSF. **Table S3.** Baseline clinical characteristics of enrolled participants. **Table S4.** Change in routine CSF parameters from baseline to after 24 weeks of treatment. **Table S5.** Safety analysis of lenalidomide. **Figure S1.** Study flow. **Figure S2.** Measurement of levels of proinflammatory cytokines in CSF. **Figure S3.** Analysis of enrichment of the genes for proinflammatory cytokines in CSF. **Figure S4.** Change in complete blood count after lenalidomide treatment.

## Data Availability

The datasets supporting the conclusions of this article are available from the corresponding author upon reasonable request.

## Abbreviations

α2M: Alpha 2-macroglobulin
Apo.AI: Apolipoprotein AI
Apo. E: Apolipoprotein E
ART: Antiretroviral therapy
Aβ42: Amyloid beta 1–42
CFH: Complement Factor H
CM: Cryptococcal meningitis
CNS: Central nervous system
CSF: Cerebrospinal fluid
FGF-2: Fibroblast growth factor-2
G-CSF: Granulocyte colony-stimulating factor
GM-CSF: Granulocyte–macrophage colony-stimulating factor
GO: Gene Ontology
GRO: Growth-related oncogene
HIV: Human immunodeficiency
HIV-CM: HIV-associated cryptococcal meningitis
IFN-γ: Interferon-γ
IgG: Immunoglobulin G
IL-1α: Interleukin-1α
IL-1β: Interleukin-1β
IL-10: Interleukin-10
IL-12p40: Interleukin-12p40
IL-12p70: Interleukin-12p70
INSTI: Integrase strand transfer inhibitors
IP-10: Interferon gamma induced protein 10
IQR: Interquartile range
MCP-1: Monocyte chemotactic protein 1
MIP-1α: Macrophage inflammatory protein-1α
MIP-1β: Macrophage inflammatory protein-1β
MRI: Magnetic resonance imaging
NNRTI: Nonnucleoside reverse transcriptase inhibitors
NRTI: Nucleoside reverse transcriptase inhibitors
PDGF-AA: Platelet-derived growth factor-AA
PI: Protease inhibitors
PLHIV: People living with HIV
pTau: Phosphorylated tau
sCD40L: Soluble CD40 ligand
Th1: T helper 1
tTau: Total tau
TNF-α: Tumor necrosis factor-α
WBC: White blood cell
