# Supplementary material for: Efficacy and safety of lenalidomide in HIV-associated cryptococcal meningitis patients with persistent intracranial inflammation: an open-label, single-arm, prospective interventional study
Source: J Neuroinflammation. 2023 Feb 15;20:38. doi: 10.1186/s12974-023-02717-w (PMC9933282; doi:10.1186/s12974-023-02717-w)
Supplement: Supplementary file 1 — Additional file 1. Definition of primary HIV infection. Diagnostic criteria for HIV-CM. Inclusion criteria. Exclusion criteria. Table S1. Follow-up procedures and items for the clinical trial. Table S2. Abbreviations for cytokines in CSF. Table S3. Baseline clinical characteristics of enrolled participants. Table S4. Change in routine CSF parameters from baseline to after 24 weeks of treatment. Table S5. Safety analysis of lenalidomide. Figure S1. Study flow. Figure S2. Measurement of levels of proinflammatory cytokines in CSF. Figure S3. Analysis of enrichment of the genes for proinflammatory cytokines in CSF. Figure S4. Change in complete blood count after lenalidomide treatment. [file 12974_2023_2717_MOESM1_ESM.docx]

**Additional file 1**

**Content**

[Definition of primary HIV infection 2](#_Toc120046564)

[Diagnostic criteria for HIV-CM 2](#_Toc120046565)

[Definition of Successful induction therapy 2](#_Toc120046566)

[Inclusion criteria 2](#_Toc120046567)

[Exclusion criteria 3](#_Toc120046568)

[Therapeutic Approaches 3](#_Toc120046569)

[Antifungal Treatment 3](#_Toc120046570)

[Lenalidomide Treatment 3](#_Toc120046571)

[Table S1 Follow-up procedures and items for the clinical trial 4](#_Toc120046572)

[Table S2 Abbreviations for cytokines in CSF 5](#_Toc120046573)

[Table S3 Baseline clinical characteristics of enrolled participants 6](#_Toc120046574)

[Table S4 Change in routine CSF parameters from baseline to after 24 weeks of treatment 7](#_Toc120046575)

[Table S5 Safety analysis of lenalidomide 8](#_Toc120046576)

[Figure S1 Study flow 9](#_Toc120046577)

[Figure S2 Measurement of levels of proinflammatory cytokines in CSF 10](#_Toc120046578)

[Figure S3 Analysis of enrichment of the genes for proinflammatory cytokines in CSF 11](#_Toc120046579)

[Figure S4 Change in complete blood count after lenalidomide treatment 12](#_Toc120046580)

# Definition of primary HIV infection

1.High-risk exposure within previous 6 weeks

2.Detectable virus in plasma (p24 Ag and/or HIV-RNA) and/or evolving anti-HIV antibody reactivity (negative or indeterminate to positive)

3.With or without clinical symptoms

4. Everyone with detectable HIV-VL and negative or indeterminate serology must receive confirmation of anti-HIV antibody seroconversion in follow-up testing. The interval of testing is one week

# Diagnostic criteria for HIV-CM

1.HIV infected patients with meningitis-related symptoms, signs, abnormal cerebrospinal fluid findings or abnormalities in intracranial imaging

2. Positive microscopy, OR positive CSF culture

# Definition of Successful induction therapy*

1. Complete 2-week induction treatment [AmB (0.7–1.0 mg/kg) + 5-FC (100 mg/kg bid)]

2. Improvement in clinical manifestations: fever headache neck stiffness, lethargy, altered mentation, elevated ICP and so on

3. Cerebrospinal fluid cryptococcal cultures were negative twice or more

*all of the above should be met simultaneously

# Inclusion criteria

1. HIV-1 infected individuals

2. Cryptococcal disease diagnosed pre-ART by positive culture or typical clinical features plus positive India ink staining or antigen detection

3. Completed induction phase therapy and consolidation treatment successfully

4. Treated with antiretroviral therapy

5. Plasma HIV-1 RNA below 500 copies per milliliter

6. Patients with chronic inflammation of the central system (presence of one or more of the following)

1. CSF protein is higher than 0.45g/L

2. CSF nucleated cell count is greater than 8/uL

3. Cranial radiological examination revealed abnormal signal lesions such as inflammation, edema and so on

7. Patients are willing to give informed consent

# Exclusion criteria

1. Pregnant or breast-feeding

2. Patients with poor treatment compliance

3. Patients who have been treated with immunosuppressants or other immunomodulators or cytotoxic drugs within 6 months before screening

4. Patients with severe cardiovascular and cerebrovascular diseases or liver and kidney dysfunction.

5. Absolute neutrophil count at 1000 cells per μL or less, platelet count less than 75000/ul

6. A known hypersensitivity or contraindication to lenalidomide

7. Participated in other clinical trials within 3 months

8. Patients with severe mental illness

# Therapeutic Approaches

## Antifungal treatment

All patients had already completed initial therapy of CM with amphotericin B (AmB) and 5-flucytosine (5-FC) and had negative CSF fungal cultures; they continued treatment with fluconazole (FLU). The induction treatment comprised AmB (0.7–1.0 mg/kg) + 5-FC (100 mg/kg bid) for 2-4 weeks. The consolidation treatment comprised 800 mg FLU for 8 weeks, while the maintenance treatment comprised 200 mg FLU daily.

## Lenalidomide treatment

Patients were treated with six cycles of lenalidomide (one cycle: 25 mg/day for 3 weeks followed by 1 week ceased lenalidomide) when they were enrolled. The drug was stopped at the end of 6 cycles.

# Table S1 Follow-up procedures and items for the clinical trial

| Time | Screened | BL 0W | 4w±7d | 8w±7d | 12w±7d | 24w±7d |
| --- | --- | --- | --- | --- | --- | --- |
| Visit point | 0 | 1 | 2 | 3 | 4 | 5 |
| Medical history | √ | √ | √ | √ | √ | √ |
| Informed consent | √ | √ |  |  |  |  |
| blood tests |  |  |  |  |  |  |
| full blood counts | √ | √ | √ | √ | √ | √ |
| liver and kidney function test | √ | √ | √ | √ | √ | √ |
| T-SPOT.TB assay | √ |  |  |  |  |  |
| CRP | √ | √ | √ | √ | √ | √ |
| coagulation function | √ | √ | √ | √ | √ | √ |
| cryptococcal antigen | √ |  |  |  |  |  |
| HIV RNA | √ |  |  |  |  | √ |
| CD4+T cell and other immune cell subsets | √ |  | √ | √ | √ | √ |
| Lumbar puncture |  | √ | √ | √ | √ | √ |
| CSF pressure |  | √ | √ | √ | √ | √ |
| Routine CSF parameters |  | √ | √ | √ | √ | √ |
| CSF biochemical analysis |  | √ | √ | √ | √ | √ |
| cytokines |  | √ | √ | √ | √ | √ |
| Imaging |  |  |  |  |  |  |
| Electrocardiogram | √ | √ | √ | √ | √ | √ |
| Cranial MRI | √ |  |  | √ |  | √ |
| chest CT scan | √ |  |  | √ |  | √ |

T-SPOT: a T-cell-based IFN-γ release assays; CRP: C-reactive protein; CSF: Cerebrospinal fluid; MRI: Magnetic resonance imaging; CT: Computed tomography;

# Table S2 Abbreviations for cytokines in CSF

| α2M | alpha 2-macroglobulin |
| --- | --- |
| Apo.AI | apolipoprotein AI |
| Apo. E | apolipoprotein E |
| Aβ42 | amyloid beta 1-42 |
| CFH | complement Factor H |
| FGF-2 | fibroblast growth factor -2 |
| G-CSF | granulocyte colony-stimulating factor |
| GM-CSF | granulocyte-macrophage colony-stimulating factor |
| GRO | growth-related oncogene |
| IFN-γ | interferon-γ |
| IL-1α | interleukin-1α |
| IL-1β | interleukin-1β |
| IL-10 | interleukin-10 |
| IL-12p40 | interleukin-12p40 |
| IL-12p70 | interleukin-12p70 |
| IP-10 | interferon gamma induced protein 10 |
| MCP-1 | monocyte chemotactic protein 1 |
| MIP-1α | macrophage inflammatory protein-1α |
| MIP-1β | macrophage inflammatory protein-1β |
| PDGF-AA | platelet-derived growth factor-AA |
| pTau | phosphorylated tau |
| sCD40L | soluble CD40 ligand |
| tTau | total tau |
| TNF-α | tumor necrosis factor-α |

| ID | Age | Gender | Race | Time from CM to ART initiation, d | Time from ART initiation to LEN, d | Time from anti antifungal to LEN, d | BMI | ART | CD4+ T cells  /ul | HIV-RNA  Copes/ml |
| --- | --- | --- | --- | --- | --- | --- | --- | --- | --- | --- |
| NO.001 | 32 | M | Asian | 26 | 196 | 222 | 24.9 | TDF+3TC+DTG | 73 | ＜20 |
| NO.002 | 22 | M | Asian | 36 | 1371 | 1407 | 20.1 | TDF+3TC+EFV | 343 | ＜20 |
| NO.003 | 39 | M | Asian | 25 | 340 | 365 | 20.2 | TDF+3TC+DTG | 132 | ＜20 |
| NO.004 | 59 | M | Asian | -14 | 1575 | 1561 | 28.0 | TDF+3TC+EFV | 199 | ＜20 |
| NO.005 | 31 | M | Asian | 25 | 623 | 648 | 26.1 | TDF+3TC+DTG | 312 | ＜20 |
| NO.006 | 29 | M | Asian | 31 | 236 | 267 | 19.3 | TDF+3TC+DTG | 102 | ＜20 |
| NO.007 | 32 | M | Asian | 38 | 525 | 563 | 21.3 | 3TC+TDF+LPV/r | 122 | 231 |
| NO.008 | 33 | M | Asian | 40 | 220 | 260 | 18.4 | TDF+3TC+DTG | 84 | 447 |
| NO.009 | 27 | M | Asian | 8 | 591 | 599 | 21.1 | TDF+3TC+DTG | 342 | ＜20 |
| NO.010 | 45 | M | Asian | 40 | 700 | 740 | 27.3 | TDF+3TC+DTG | 359 | 448 |
| NO.011 | 32 | M | Asian | -14 | 492 | 478 | 25.6 | TAF+FTC+BIC | 447 | ＜20 |
| NO.012 | 40 | M | Asian | 22 | 1303 | 1325 | 26.0 | TDF+3TC+RAL | 122 | ＜20 |
| NO.013 | 39 | M | Asian | 25 | 742 | 767 | 20.2 | TDF+3TC+DTG | 131 | ＜20 |
| NO.014 | 35 | M | Asian | 35 | 356 | 391 | 28.4 | TDF+3TC+DTG | 171 | ＜20 |

# Table S3 Baseline clinical characteristics of enrolled participants

ART: antiretroviral therapy; BMI: Body Mass Index; CM: cryptococcal meningitis; LEN: Lenalidomide; d: days

# Table S4 Change in routine CSF parameters from baseline to after 24 weeks of treatment

| Time | Week 0 | Week 4 | Week 8 | Week 12 | Week 24 |
| --- | --- | --- | --- | --- | --- |
| ICP (mmH_2_O) | 150.0 (135.0-210.0) | 160.0(144.0-220.0) | 130 (97.5-215.0) | 200 (123.8-210.0) | 150.0 (120.00-175.0) |
| CSF WBC (10^6^/L) | 35.0 (4.5-90.0) | 3.0 (0-40.0) * | 8.5 (4.3-12.0) ∆ | 10.0 (2.0-24.3) & | 10.0 (2.0-14.0) # |
| CSF chloride (mmol/L) | 123.5 (118.8-127.3) | 126.0 (120.0-127.0) | 122.5 (119.3-125.5) | 124.0 (123.0-126.0) | 126.0 (121.0-128.0) |
| CSF glucose (mmol/L) | 2.6 (2.1-2.9) | 2.5 (2.3-2.8) | 2.5 (2.4-2.7) | 2.7 (2.5-3.0) | 2.70 (2.5-2.8) # |
| CSF protein (g/L) | 1.4 (0.7-3.2) | 0.9 (0.7-1.2) * | 0.8 (0.6-1.1) ∆ | 0.7 (0.5-0.9) & | 0.8 (0.5-1.1) # |
| CSF ALB (mg/L) | 79.2 (48.4-149.8) | 55.3 (39.3-78.5) * | 46.1 (37.0-62.1) ∆ | 42.8 (24.2-94.8) & | 42.4 (32.7-54.4) # |
| CSF IgG (mg/dL) | 13.1 (7.3-160.5) | 13.9 (9.4-47.3) | 13.3 (6.3-35.7) | 10.5 (5.0-33.5) | 11.2 (6.4-36.0) |

P value was determined by the Wilcoxon matched-pairs signed rank test. *W4 vs W0, P< 0.05; ∆W8 vs W0, P< 0.05; &W12 vs W0 , P< 0.05; #W24 vs W0, P< 0.05. ICP: intracranial pressure; CSF: cerebrospinal fluid；WBC: white blood cells；ALB: albumin; IgG: Immunoglobulin G

# Table S5 Safety analysis of lenalidomide

| Follow-up | Week 0 | Week 24 | P Value* |
| --- | --- | --- | --- |
| Routine blood test |  |  |  |
| HB (g/L) | 134.0 (130.0-138.0) | 132.0 (131.0-145.0) | 0.720 |
| WBC (10E9/L) | 6.8 (5.7-8.1) | 4.30 (3.6-4.6) | **0.042** |
| N (10E9/L) | 3.8 (2.9 -4.8) | 1.3 (1.2-2.0) | **0.019** |
| PLT (10E9/L) | 260.0 (209.0-299.0) | 198.0 (187.0-210.0) | **0.010** |
| E (10E9/L) | 0.20 (0.15-0.32) | 0.18（0.06-0.26） | 0.067 |
| Biochemistry |  |  |  |
| ALT (U/L) | 19.0 (18.0-27.0) | 19.0(18.0-27.0) | 0.285 |
| AST(U/L) | 15.0 (10.5-19.5) | 17.0(14.0-21.0) | 0.305 |
| TB (umol/L） | 5.8 (3.7-7.2) | 8.3 (4.9-10.3) | **0.020** |
| ALB (g/L) | 41.5 (40.1-42.5) | 41.7(39.9-42.8) | 0.520 |
| Cr (umol/L） | 84.5 (80.3-97.3) | 90.0 (87.0-96.0) | 0.722 |
| BUN (umol/L） | 4.60 (3.6-4.9) | 5.3(4.3-5.6) | **0.010** |
| TG (mmol/L） | 1.8 (1.4-3.2) | 2.3 (1.5-2.6) | 0.859 |
| TC (mmol/L） | 3.60 (3.3-4.5) | 4.0 (3.5-4.2) | 0.831 |
| CRP (mg/ml） | 3.4 (1.6-7.9） | 3.2 (1.4-4.7) | 0.465 |
| Coagulation function |  |  |  |
| PT (S) | 11.4 (10.9-11.7) | 11.200 (10.9 -11.5) | 0.483 |
| D-dimer | 299.0 (185.0-505.0) | 169.0 (169.0-207.0) | **0.009** |
| CD4+T cell (cells/ul) | 151.5 (117.0-342.3) | 202.0 (153.5-307.5) | 0.102 |
| CD8+T cell (cells/ul) | 1038.0 (724.0-1355.0) | 989.0 (896.0-1289.0) | 0.638 |

*P value was determined by the Wilcoxon matched-pairs signed-rank test. HB: hemoglobin; WBC: white blood cells; N: neutrophils; PLT: platelet; E: eosinophil; ATL: alanine aminotransferase; AST: aspartate aminotransferase; TB: total bilirubin; ALB: albumin; Cr: creatinine; BUN: blood urea nitrogen; TG: triglyceride; TC: total cholesterol; CRP: c-reactive protein; PT: prothrombin time

# Figure S1 Study flow


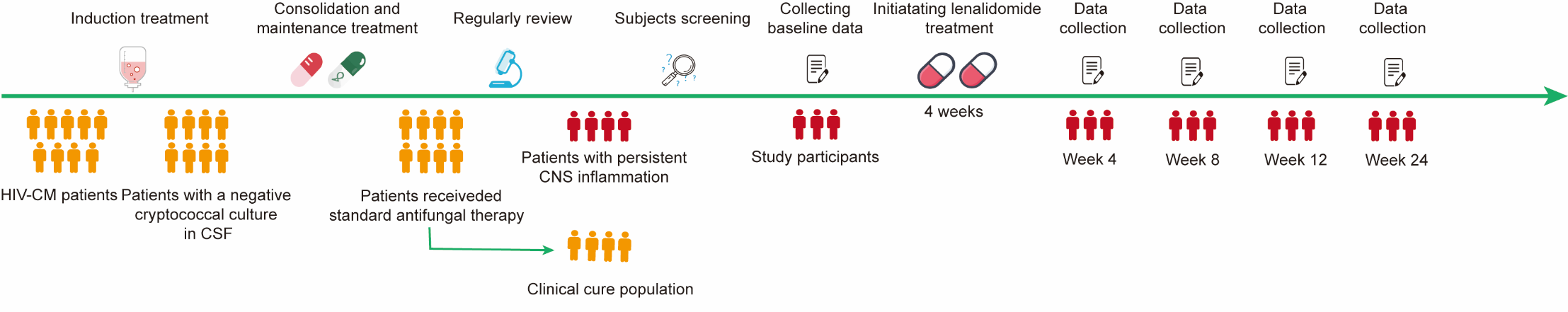


Patients enrolled in this study were HIV-1-infected individuals with CM. All patients had already completed initial therapy of CM with amphotericin B (AmB) and 5-flucytosine (5-FC) and had negative CSF fungal cultures; they continued consolidation treatment and maintenance treatment with fluconazole (FLU). Received standard antifungal and antiretroviral therapy, these patients had a negative cryptococcal culture in CSF. However, CSF indices and neuroimaging characteristics remained abnormal, and clinical manifestations did not improve significantly (or even worsened), which was thought to be attributed to persistent intracranial inflammation. Based on the inclusion and exclusion criteria, we screened the patients and finally tried to treat them with lenalidomide. Final participants received five visits over 24 weeks (baseline as well as at weeks 4, 8, 12, and 24). At these visits, we assessed clinical symptoms and adverse reactions, and undertook blood tests, clinical data and samples collection. CSF: Cerebrospinal fluid; CNS: Central Nervous System; HIV-CM: HIV-associated cryptococcal meningitis

#
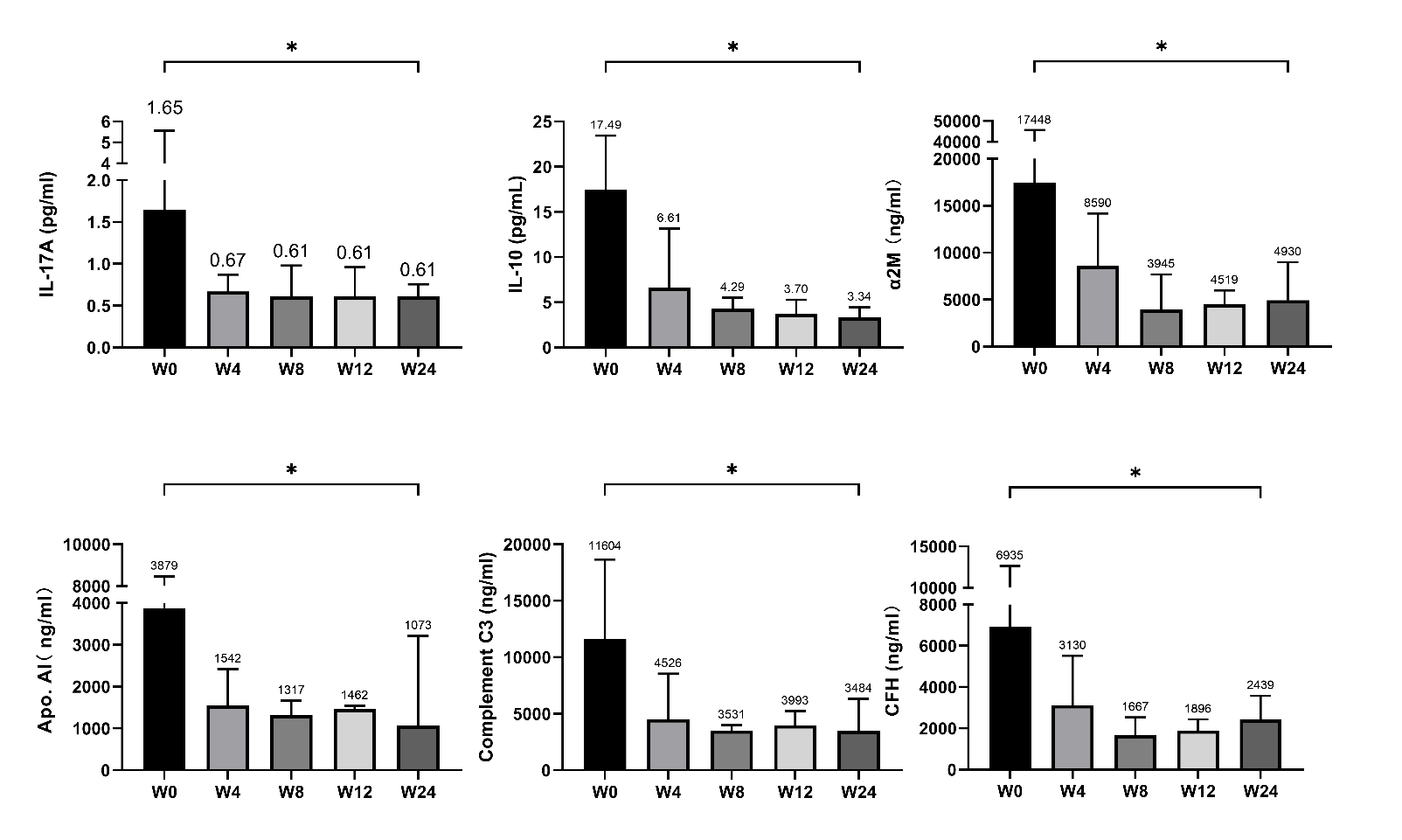
Figure S2 Measurement of levels of proinflammatory cytokines in CSF

Concentrations of IL-17A, IL-10, α2M, Apo.AI, complement C3, CFH in the CSF from patients decrease significantly after treating with lenalidomide for 24 weeks. CSF cytokines profile data at week [W] 24 and W0 were compared using the Wilcoxon matched-pairs signed-rank test. * W24 vs W0, P< 0.05. IL: interleukin; α2M: alpha 2-macroglobulin; Apo.AI: apolipoprotein AI; CFH: complement Factor H

#
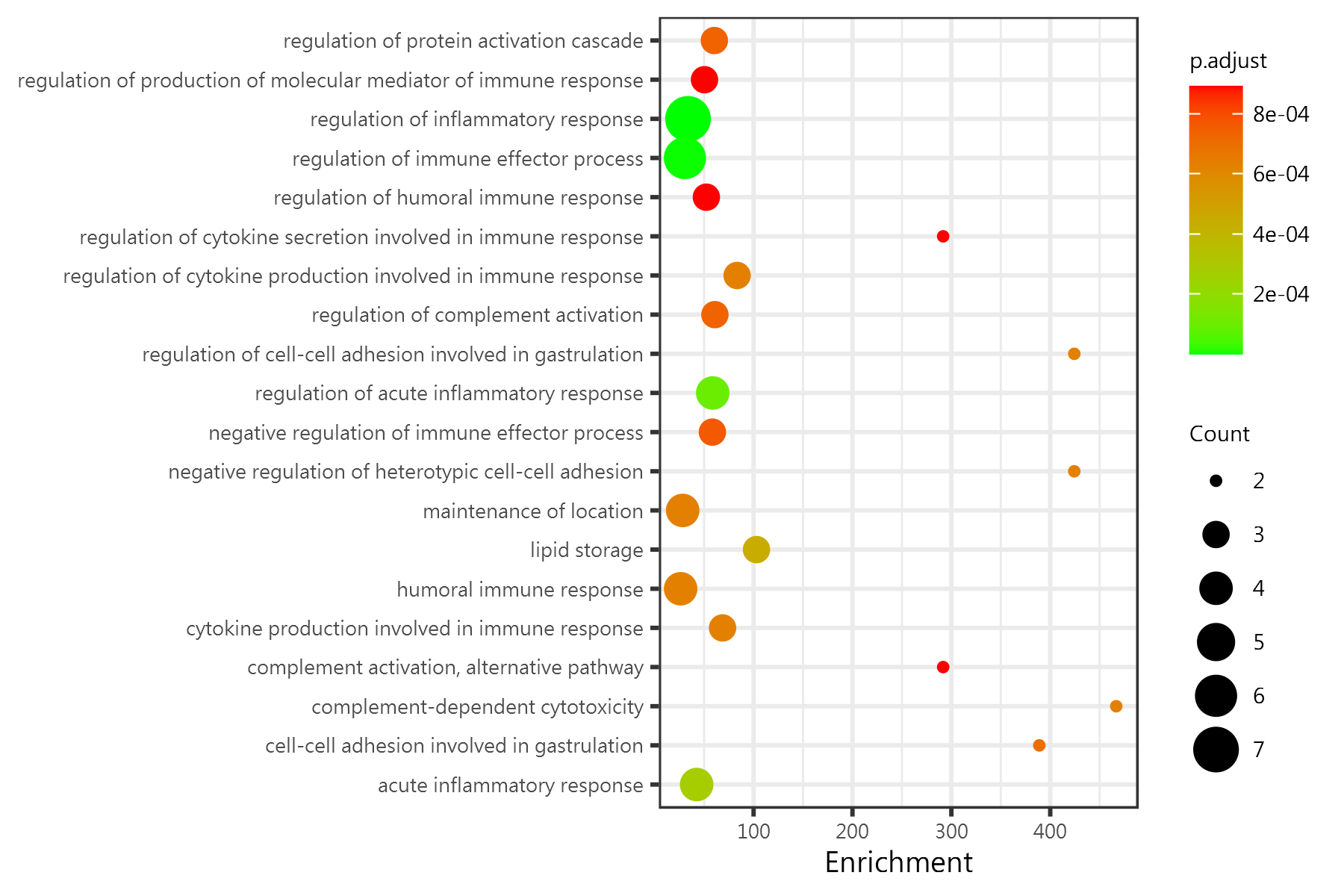
Figure S3 Analysis of enrichment of the genes for proinflammatory cytokines in CSF

#
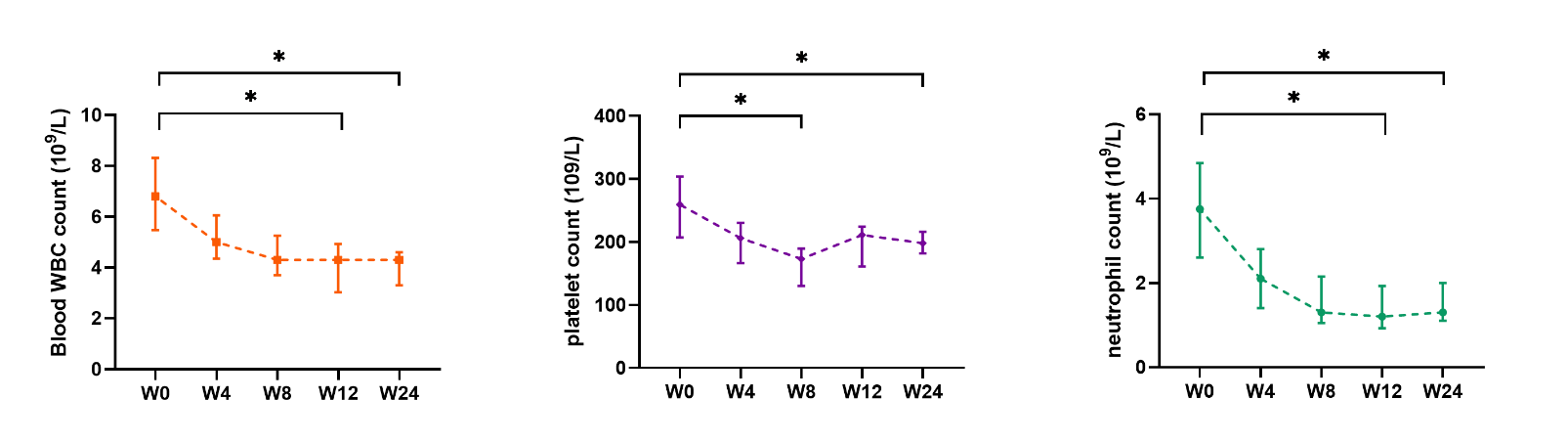
Figure S4 Change in complete blood count after lenalidomide treatment

WBC: white blood cells;**P<0.01
